# Physiology of the volume-sensitive/regulatory anion channel VSOR/VRAC. Part 1: from its discovery and phenotype characterization to the molecular entity identification

**DOI:** 10.1186/s12576-023-00897-x

**Published:** 2024-01-18

**Authors:** Yasunobu Okada

**Affiliations:** 1 National Institute for Physiological Sciences, 5-1 Higashiyama, Myodaiji, Okazaki, Aichi 444-8787 Japan; 2 Department of Integrative Physiology, Graduate School of Medicine, Akita University, Akita, Japan; 3 Department of Physiology, School of Medicine, Aichi Medical University, Nagakute, Japan; 4 Department of Physiology, Kyoto Prefectural University of Medicine, Kyoto, Japan; 5 Cardiovascular Research Institute, Yokohama City University, Yokohama, Japan; 6 Graduate University for Advanced Studies (SOKENDAI), Hayama, Kanagawa Japan

**Keywords:** Volume-sensitive anion channel, Regulatory volume decrease, Phenotypical properties, Molecular entities, LRRC8, TRPM7

## Abstract

The volume-sensitive outwardly rectifying or volume-regulated anion channel, VSOR/VRAC, which was discovered in 1988, is expressed in most vertebrate cell types and is essentially involved in cell volume regulation after swelling and in the induction of cell death. This series of review articles describes what is already known and what remains to be uncovered about the functional and molecular properties as well as the physiological and pathophysiological roles of VSOR/VRAC. This Part 1 review article describes, from the physiological standpoint, first its discovery and significance in cell volume regulation, second its phenotypical properties, and third its molecular identification. Although the pore-forming core molecules and the volume-sensing subcomponent of VSOR/VRAC were identified as LRRC8 members and TRPM7 in 2014 and 2021, respectively, it is stressed that the identification of the molecular entity of VSOR/VRAC is still not complete enough to explain the full set of phenotypical properties.

## Background

In animal cells, on which the rigid cell wall is lacking, cell volume regulation is essential for their survival and functions. Even under extracellular hypoosmotic or intracellular hyperosmotic conditions, the cells exhibit the regulatory volume decrease (RVD) shortly after osmotic swelling. In 1988, the RVD process was, for the first time, demonstrated to be attained by water efflux driven by KCl efflux due to parallel activation of K^+^ channels and Cl^−^ channels by Hazama & Okada [1] and independently by Cahalan & Lewis [2]. Although the volume-regulatory K^+^ channel in epithelial cells was shown to be directly activated by an increase in the intracellular Ca^2+^ concentration and is thus classified into a well-known Ca^2+^-activated K^+^ channel, the Cl^−^ channel was demonstrated to be not directly activated by cytosolic Ca^2+^ rise, unlike the Ca^2+^-activated Cl^−^ channel (CaCC), and to be a new type of anion channel [1]. This swelling-activated anion channel is expressed in most vertebrate cell types studied to date and is nowadays called the volume-sensitive outwardly rectifying anion channel (VSOR: [3]) or the volume-regulated/regulatory anion channel (VRAC: [4]). The volume-sensitive/regulatory outwardly rectifying anion channel, here called VSOR/VRAC, was then discovered to play an essential role also in the induction of apoptotic cell death [5, 6] and cisplatin resistance in cancer cells [7]. Thereafter, in 2014, LRRC8A was identified as the prerequisite molecule of VSOR/VRAC [8, 9], and the heteromer of LRRC8A and the other member(s) of LRRC8 (LRRC8C/D/E) was shown to exert as the core component of VSOR/VRAC channels [9]. Furthermore, the volume expansion sensitivity of VSOR/VRAC was recently shown to be granted by mechano-sensitive Ca^2+^-permeable cation channel TRPM7 which serves as the essential subcomponent of VSOR/VRAC channels by physically interacting with LRRC8A [10]. However, from the standpoint of the phenotypical properties of VSOR/VRAC, there is still a missing subcomponent for the molecular entity of VSOR/VRAC [11]. As the 35th anniversary of the VSOR/VRAC discovery, this review article describes the roles in cell volume regulation, the phenotypical properties, and the molecular entities, including its pore-forming core component and volume-sensing subcomponent, of VSOR/VRAC with evaluating what is already elucidated and what remains unknown mainly from the physiological standpoint.

## Introduction: cell volume regulation and discovery of VSOR/VRAC

All animal cells have appropriate cell volume, and cell volume regulation is essential for their survival (see Reviews: [12–14]; also see Books: [15, 16]).

### Cell volume perturbation

**Fig. 1:**
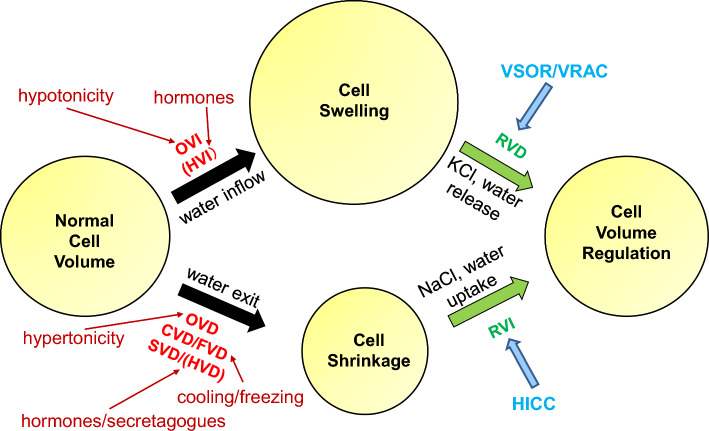
Cell volume changes (shrinkage and swelling) caused by osmotic perturbation and cell volume regulation thereafter. Osmotic volume decrease (OVD), secretory volume decrease (SVD), and cooling/freezing volume decrease (CVD/FVD) are shortly followed by the regulatory volume increase (RVI), whereas osmotic volume increase (OVI) is by the regulatory volume decrease (RVD). Hormonal volume decrease (HVD) and increase (HVI) are given in parenthesis, because it is not known whether they are followed by RVI and RVD, respectively. The key players for RVI and RVD are cationic HICC channels and anionic VSOR/VRAC channels, respectively. (See text for details.)

Although body fluid osmolarity is kept constant (within 1–2%) by the thirst-antidiuretic hormone mechanisms, significant changes in local osmolarity are, even under physiological conditions, elicited around cells (see Review: [11]). Under pathological conditions, sizable changes in the plasma osmolarity are produced in association with a variety of diseases (see Table 1 in [17]). Upon such osmotic perturbation, the volume of animal cells is forced to be altered (Fig. 1) for the following reasons: First, the cell membrane exhibits high water permeability (up to 100 μm/s) and thus behaves as a semipermeable membrane. Second, animal cells, unlike plant cells, do not have a rigid cell wall. Thus, as illustrated in Fig. 1, the cells are rendered to rapidly exhibit osmotic shrinkage or an osmotic volume decrease (OVD) and swelling or an osmotic volume increase (OVI) in response to a hypertonic challenge induced by extracellular hypertonicity or intracellular hypotonicity and a hypotonic challenge by extracellular hypotonicity or intracellular hypertonicity, respectively [17]. In addition, in response to stimulation with secretagogues, secretory cells exhibit shrinkage, called the secretory volume decrease (SVD) [20]. Upon cooling and freezing, cells also show shrinkage, called the cooling and freezing volume decrease (CVD and FVD) [21]. A variety of hormones are known to induce cell volume changes by activating electroneutral ion cotransporters (antiporters and symporters) or by stimulating intracellular metabolism (see Table 2 in [18]). Thus, we term volume changes produced by such hormonal stimulation as the hormonal volume decrease (HVD) and increase (HVI). The most well-known HVD- and HVI-inducing hormones are insulin and glucagon, respectively, in hepatocytes [19]. Although HVD/HVI/SVD and CVD/FVD are both to be, in the broad sense, classified into osmotic volume changes, the former is caused by primary changes in the osmolyte concentrations whereas the latter is by primary changes in the activity of water.

**Table 1:** Phenotypes of the properties of VSOR/VRAC

A. Physiological properties	
Cell volume sensitivity	Activation by cell swelling (volume expansion) but not directly by mechano-stress
Cytosolic ATP dependence	Non-hydrolytic requirement of intracellular ATP
Cytosolic Mg^2+^ sensitivity	Sensitivity to intracellular free Mg^2+^
B. Biophysical properties	
Single-channel level	Intermediate outwardly rectifying unitary conductance under symmetrical Cl^−^ conditions : 10–40 pS at negative potentials 30–80 pS at positive potentials at physiological Cl^−^ concentrations
Whole-cell current level	
Voltage dependence	Moderate outward rectification Inactivation kinetics at large positive potentials Open-channel blocking by extracellular ATP
Anion selectivity	Low-field anion selectivity: Eisenman type I sequence : I^−^ > Br^−^ > Cl^−^

**Table 2:** Physicochemical Halide Anion Selectivity Sequence

Eisenman type	Sequence	Corresponding anion channel
I (low-field)	I^−^ > Br^−^ > Cl^−^ > F^−^	VSOR/VRAC, ASOR/PAC, CaCC, Maxi-Cl
II	Br^−^ > I^−^ > Cl^−^ > F^−^	
III	Br^−^ > Cl^−^ > I^−^ > F^−^	CFTR
IV	Cl^−^ > Br^−^ > I^−^ > F^−^	ClC-2
V	Cl^−^ > Br^−^ > F^−^ > I^−^	
VI	Cl^−^ > F^−^ > Br^−^ > I^−^	
VII (strong-field)	F^−^ > Cl^−^ > Br^−^ > I^−^	

VSOR/VRAC represents the volume-sensitive outwardly rectifying/volume-regulatory anion channel, ASOR/PAC the acid-sensitive outwardly rectifying/proton-activated anion channel, CaCC the Ca^2+^-activated chloride channel, Maxi-Cl the large-conductance maxi-anion channel, CFTR the cyclic AMP-activated cystic fibrosis transmembrane conductance regulator anion channel, and ClC-2 the voltage-gated chloride channel-2

### Cell volume regulation and swelling-activated anion channels

Shortly after cell shrinkage (OVD/SVD/CVD/FVD) and swelling (OVI) forced by non-steady state osmotic perturbation, animal cells can regulate their volume by the mechanisms called the regulatory volume increase and decrease (RVI and RVD) due to water flux mainly driven by uptake of NaCl and release of KCl, respectively [5–7] (Fig. 1). However, it is not known whether HVD and HVI are followed by regulatory volume responses. It is noteworthy that arginine vasopressin (AVP) neurons were recently demonstrated to respond to hyperosmotic stimulation not only with osmotic shrinkage (OVD) followed by RVI but also with persistent cell shrinkage (SVD) due to massive AVP release in an additive manner by Sato-Numata et al. [22]. These volume-regulatory NaCl and KCl transports had long been considered to be attained by the operation of electroneutral ion coupled transporters such as the Na^+^-K^+^-Cl^−^ symporter or cotransporter (NKCC), Na^+^-Cl^−^ symporter (NCC), or parallel operation of Na^+^/H^+^ antiporter or exchanger (NHE) *plus* Cl^−^/HCO_3_^–^ antiporter (anion exchanger: AE) and K^+^-Cl^−^ symporter (KCC) or parallel operation of K^+^/H^+^ antiporter (KHE) *plus* AE, respectively (see Reviews: [23, 24]. However, it is now established that these volume-regulatory fluxes of Na^+^, K^+^, Cl^−^, and small negatively-charged organic solutes are predominantly due to the activation of a number of channel-mediated conductive pathways in most cell types. Among them, the main actor for RVI is a shrinkage-activated Na^+^-permeable cation channel which was, for the first time, observed in 1989 [25] and is nowadays called the hypertonicity-induced cation channel (HICC: [26]; also see Review [27]) (Fig. 1). Meanwhile, the key player for RVD is a swelling-activated anion channel which was originally observed in 1988 by Hazama & Okada in human epithelial Intestine 407 cells [1, 28] and independently by Cahalan & Lewis in human lymphoid cells [2], and is nowadays called the volume-sensitive outwardly rectifying anion channel (VSOR: [3]) or the volume-regulated anion channel (VRAC: [4]) (Fig. 1). These two groups also, for the first time, independently demonstrated that parallel activation of VSOR/VRAC Cl^−^ channels and K^+^ channels is responsible for the attainment of RVD [1, 2].

In the present Part 1 article, I review the properties and molecular entities of VSOR/VRAC channels from the physiological standpoint. The activation mechanisms and the roles in the transport of organic signal molecules, cell death induction, and anticancer drug resistance of VSOR/VRAC are to be reviewed in the subsequent Part 2 article.

## Phenotypical properties of VSOR/VRAC

Since the discovery of VSOR/VRAC, its biophysical and physiological properties have been extensively investigated and well summarized in review articles in the early period [3, 4, 29] and in the last few years [30–35] with including information accumulated on the basis of the recent identification of LRRC8 as the core molecule of VSOR/VRAC [8, 9]. The pharmacological properties of VSOR/VRAC have also been extensively studied, as summarized in review articles [3, 4, 32, 34, 36, 37]. Here, these properties are updated by taking the latest studies into consideration.

### The physiological and biophysical properties

The phenotypical or physiological and biophysical properties of VSOR/VRAC, which are distinct from other types of anion channels [34], are summarized in Table 1 (A and B, respectively) as the starting or basic point for ongoing and future studies.

#### Cell volume sensitivity

Cell swelling-induced activation is the most important property of VSOR/VRAC, and this is not directly elicited by membrane stretch, because activation of VSOR/VRAC per se was never elicited by the suction of cell-attached patch membranes [3, 38–40]. Under physiological and quasi-physiological conditions, moderate cell swelling (volume expansion) may take place without producing drastic plasma membrane stretch because the cell membrane has, in general, sufficient reserves due to the existence of its infoldings [41, 42]. In fact, the cell surface area estimated from the whole-cell membrane capacitance of human epithelial Intestine 407 cells was more than four-fold larger than that measured by image analysis [43]. Since unitary VSOR/VRAC events could be recorded only when the patch pipette had been attached to the cell after, but never before, the cell was rendered swelling [3, 44], membrane expansion mainly due to unfolding of the invagination is likely to be required for VSOR/VRAC activation. As a matter of fact, human epidermoid KB cells exhibited a nearly linear relationship between the VSOR/VRAC current density and the relative surface area in a certain range above the threshold [45]. Also, it is noteworthy that VSOR/VRAC currents can be activated by cell inflation (swelling) induced by the injection of iso-osmotic solution with identical ionic strength into the cells under whole-cell configuration [46–48].

#### Cytosolic ATP dependence and Mg^2+^ sensitivity

The presence of intracellular ATP is a prerequisite to VSOR/VRAC activity. This fact was, for the first time, reported by two groups in mouse T lymphocytes [49] and in human epithelial HeLa cells [50]. Independently, such intracellular ATP dependence of VSOR/VRAC was subsequently reported by three groups in rat glioma C6 cells [51], human umbilical endothelial cells [52], and human Intestine 407 cells [53]. Our recent quantitative studies in Intestine 407 cells showed that the half-maximal effective concentration (EC_50_) for ATP-induced VSOR/VRAC activation was 14 μM in the presence of 1 mM free Mg^2+^ (Fig. 3 in [34]). Moreover, it was found that VSOR/VRAC activity requires ATP but not its hydrolysis because such an ATP role can be substituted by non-hydrolysable ATP analogs [50–54]. This fact suggests an essential role of some ATP-binding protein(s) in the mechanism of VSOR/VRAC activation. On the other hand, intracellular free Mg^2+^ was found to suppress VSOR/VRAC activity [53, 55]. The half-maximal inhibitory concentration (IC_50_) for free Mg^2+^ was 0.32 and 1.9 mM in the presence of 0.01 and 0.1 mM free ATP, respectively [34]. Such free ATP requirement and free Mg^2+^ sensitivity suggest that free Mg^2+^ inhibits VSOR/VRAC channels by forming the Mg-ATP complex.

#### Intermediate outwardly rectifying unitary conductance

Single-channel recordings of VSOR/VRAC-like intermediate unitary events were, for the first time, observed in human airway epithelial cells [56] and colonic epithelial T84 cells [57]. Subsequently, we provided firm evidence that such intermediate unitary events, in fact, represent VSOR/VRAC activity by performing the double patch-clamp recording, which can simultaneously observe single-channel currents and whole-cell currents, in swollen human Intestine 407 cells [38, 58] and in mouse cortical neurons [59]. Thereafter, intermediate-conductance VSOR/VRAC unitary activities were also observed in a variety of cell types including mouse mammary gland C127 cells [60], mouse thymocytes [61], and mouse cardiomyocytes in primary culture [62].

#### Three types of voltage dependence

Moderate (but not strong) outward rectification and inactivation kinetics of macroscopic whole-cell VSOR/VRAC currents are distinct from those of other types of anion channels, such as voltage-gated inwardly rectifying ClC-2, large-conductance ohmic (non-rectifying) Maxi-Cl, cAMP-activated ohmic CFTR, acid-sensitive strongly outwardly rectifying anion channel (ASOR) or proton-activated anion channel (PAC), and sharp outward-rectifier Ca^2+^-activated CaCC [34]. Our double-patch clamp recordings demonstrated that outward rectification observed even under symmetrical Cl^−^ conditions and time-dependent inactivation of outward currents observed only at large positive potentials on the macroscopic whole-cell level are caused by voltage-dependent changes in the single-channel conductance and voltage-dependent stepwise unitary closing events, respectively [38, 59, 63, 64]. Such two types of voltage dependence have been observed by many laboratories in a wide variety of cell types, including human epithelial Intestine 407 [38, 58, 65–68], T84 [68], HEK293 [69–71], and HeLa [6, 72] cells, human epidermal KB and its derivative cells [7, 45, 73], mouse mammary epithelial C127 [60, 74, 75], mouse cortical neurons [59, 63, 76], rat vasopressin (AVP) neurons [77], mouse astrocytes [78–81], mouse cardiomyocytes [82], mouse adipocytes [83], rat thymocytes [84], and chicken B lymphocyte DT-40 cells [10] so far studied in our own and collaborative laboratories. The third type of voltage dependence of VSOR/VRAC currents is the open-channel blocking by extracellular ATP upon depolarization [85–90].

#### Low-field anion selectivity

The halide anion selectivity sequence (Table 2) is determined by the product of the ratio of equilibrium constant for binding to the positively-charged binding site and the ratio of mobility between two anion species, and it is classified into the high-field strength one dominated by the former ratio and the low-field strength one dominated by the latter ratio, as described in detail by Wright and Diamond [91]. The low-field-strength halide anion selectivity with Eisenman sequence type I (I^−^  > Br^−^  > Cl^−^  > F^−^) of VSOR/VRAC is distinct from the higher-field anion selectivity of CFTR (Eisenman type III: Br^−^  > Cl^−^  > I^−^  > F^−^) and ClC-2 (Eisenman type IV: Cl^−^  > Br^−^  > I^−^  > F^−^) [34]. In our laboratory, this type of anion selectivity was confirmed for VSOR/VRAC currents in human Intestine 407 [66] and KB [73] cells as well as mouse cortical neurons [59] and rat AVP neurons [77].

#### Severe hypotonicity-activated anion currents

There were several studies suggesting intracellular ATP-independent activation of VSOR/VRAC induced by unphysiologically severe hypotonic stimulation. Jirsch et al. [92] showed that intracellular ATP removal abolished swelling-activated Cl^−^ currents (I_Cl,swell_) caused by exposure to 72% hypotonic solution but not those induced by a 55% hypotonic challenge in human H69AR cancer cells. However, it must be noted that the employed condition was just a nominally ATP-free one (without precluding contamination of residual endogenous ATP), and moreover that these currents were also evoked by suction of the cell-attached patch pipette; that is, mechano-sensitive. Next, Volk et al. [93] and Bond et al. [94] reported that intracellular ATP depletion by using metabolic inhibitors abolished I_Cl,swell_ activation induced by a modest (83–86%) hypotonic challenge but failed to abolish I_Cl,swell_ activation induced by a severe (50–73%) hypotonic challenge in rat kidney IMCD cells and mouse neuroblastoma N1E115 cells, respectively. However, it is not known whether severe hypotonicity-activated, intracellular ATP-independent I_Cl,swell_ exhibited the full set of phenotypes of VSOR/VRAC properties (Table 1) other than intracellular ATP dependence.

### Pharmacological properties

None of the VSOR/VRAC blockers are unfortunately selective, as summarized in recent review articles [32, 34, 36, 37]. Most of the VSOR/VRAC blockers can be classified into three groups (Table 3): (1) Conventional type of Cl^−^ channel blockers with two aromatic rings connected with a chain of one to four atoms (see Fig. 9B in [34]); (2) ethacrynic acid derivatives possessing the 2,3-dichloro phenoxyl fragment (see Fig. 9A in [34]); and (3) open-channel blockers.

**Table 3:** Pharmacological properties of VSOR/VRAC

Broad specificity	Sensitivity to conventional type of Cl^−^ channel blockers with two aromatic rings connected with a chain of several atoms
	Sensitivity to etacrynic acid derivatives with a 2,3-dichlorophenoxyl fragment
Open-channel blocking	Depolarization-induced blocking by the pore-plugging action of anionic chemicals from the extracellular side

#### Conventional VSOR/VRAC blockers

The first group includes a large variety of conventional Cl^−^ channel blockers such as phloretin, SITS, DIDS, NPPB, DPC, niflumic acid, flufenamic acid, NS3728, and glibenclamide. The sensitivity of VSOR/VRAC to the stilbene-derivatives, SITS and DIDS, was systematically demonstrated by Kubo & Okada [65] in human Intestine 407 cells and by Nilius et al. [95] in human umbilical endothelial cells. Blocking efficacy at positive voltages was more prominent than that at negative voltages. Such voltage-dependent VSOR/VRAC sensitivity to SITS/DIDS was confirmed in a wide variety of cell types including mouse cortical neurons [59], human KB and its derivative cells [7, 73], mouse astrocytes [79], and mouse cardiomyocytes [62, 82]. A sulfonylurea, glibenclamide, also exhibited a similar voltage-dependent blocking action to VSOR/VRAC currents in human Intestine 407 cells [96], mouse C127 cells [74], and mouse adipocytes [83]. In contrast, VSOR/VRAC currents were found to be inhibited, in a voltage-independent manner, by carboxylate analogs such as NPPB, 9-AC, and DPC as well as DPC analogs, niflumic acid and flufenamic acid, in a large variety of cell types (see Review [3]) including human Intestine 407 cells [65], HeLa cells [72], KB and its derivative cells [7], mouse cortical neurons [59, 76], rat AVP neurons [77], and mouse adipocytes [83]. A bisphenol, phloretin, was also found to voltage-independently block VSOR/VRAC activity in human colonic epithelial T84 and Intestine 407 cells as well as in mouse C127 cells [68]. Later, similar phloretin sensitivity of VSOR/VRAC was observed in mouse cortical neurons [59, 76], human KB and its derivative cells [7, 73], rat AVP neurons [77], mouse astrocytes [78, 79], and rat thymocytes [84]. Recently, a number of flavonoids, which also have two connected aromatic rings, were reported to inhibit VSOR/VRAC currents [97, 98]. In addition, novel two VSOR/VRAC blockers, pranlukast and zafirlukast, were discovered by a high-throughput screening combined with a YFP quenching assay [99]. These cysteinyl leukotriene 1 (CysLT1) receptor antagonists voltage-independently blocked VSOR/VRAC currents at micromolar concentrations in a manner independent of CysLT1. It is noted that both pranlukast and zafirlukast can also be classified into the two aromatic ring-connected conventional Cl^−^ channel blockers (RZ Sabirov: personal communication). However, the selectivity of pranlukast and zafirlukast to VSOR/VRAC against other ion channels is not known as yet.

#### Ethacrynic acid-derivative VSOR/VRAC blockers

The second ethacrynic acid-derivative group includes DCPIB, IAA-94, and DIOA. DCPIB was originally found to effectively block VSOR/VRAC currents in a voltage-independent manner in calf endothelial cells [100]. Since then, this indanone compound has been widely employed as a most selective blocker for VSOR/VRAC in many cell types including human epithelial HEK293 [70] and HeLa [72] cells, human epidermoid KB cells [7], rat AVP neurons [77], mouse astrocytes [79–81], rat thymocytes [84], and chicken B-lymphocyte DT40 cells [10]. In contrast, DCPIB was shown to be ineffective in blocking CaCC, CFTR, and ClC-1/3/4/5/K1 Cl^−^ channel activities [100] as well as Maxi-Cl [101, 102] and ASOR/PAC [72] anion channel activities. However, it must be noted that even DCPIB exhibits off-target effects on a variety of other ion channels and transporters, as summarized in review articles [34, 37, 99, 103]. In addition, one also needs to bear the possibility in mind that DCPIB may suppress, to a certain extent, some molecularly unidentified Ca^2+^-activated Cl^−^ currents [104] and acid-activated Cl^−^ currents [105]. IAA-94 was shown to inhibit VSOR/VRAC currents in mouse cortical neurons [76] and rat pyramidal neurons [106]. DIOA was also found to be an effective VSOR/VRAC blocker in microglial BV-2 cells [107] and epithelial HeLa cells [72]. However, DIOA was observed to block not only VSOR/VRAC currents but also ASOR/PAC currents in HeLa cells [72].

#### Open-channel VSOR/VRAC blockers

The third group is the open-channel blockers that exhibit depolarization-induced suppression of VSOR/VRAC currents only from the extracellular side. As noted above, extracellular free ATP^4−^ was shown to exhibit open-channel blocking actions to VSOR/VRAC currents in a variety of cell types [85–90]. Similar open-channel blocking effects were also observed in some of the above conventional Cl^−^ channel blockers. For example, SITS/DIDS and glibenclamide were found to exhibit voltage-dependent blocking action on VSOR/VRAC currents originally by Kubo & Okada [65] and Liu et al. [96], respectively. Suramin^6−^ was also shown to be an open-channel blocker of VSOR/VRAC [64, 72, 90, 108–111]. The open-channel blocking actions are very interesting from the viewpoint of the pore size estimation. In this regard, the VSOR/VRAC channel should have an outer vestibule larger and a pore smaller than the sizes of ATP, SITS, glibenclamide, and suramin the unhydrated radii of which are approximately 0.58, 0.55, 0.60, and 0.91 nm (see Fig. 15.8 in [64]), respectively. In fact, the effective pore radius was estimated to be 0.57–0.71 nm through the experiments with permeant blockers [108, 112] and around 0.63 nm by the non-electrolyte partitioning method [67].

#### Other types of VSOR/VRAC blockers

VSOR/VRAC activity was also sensitive to a large variety of other structurally unrelated chemicals (see Review [34]) including one of essential fatty acid, arachidonic acid [65], and a tyrosine kinase inhibitor, genistein [113]. Very recently, dicumarol, which is a known competitive inhibitor of vitamin K epoxide reductase, was shown to be a novel potent VSOR/VRAC inhibitor selective against ASOR and CaCC [114], However, possible off-target effects on other ion channels remain to be examined.

## Molecular entities of VSOR/VRAC

Although the phenotypical properties of VSOR/VRAC were well elucidated soon after the functional discovery of VSOR/VRAC in 1988 [1, 2], it took over a quarter of a century to identify LRRC8A, also called SWELL1, as the core component of VSOR/VRAC molecule(s) [8, 9]. In the meantime, there were long-time struggles against many false-positive candidate molecules, as summarized in recent review articles [11, 31, 32]. Among them, the biggest-hyped ones are P-glycoprotein (PGP) proposed in 1992 [115], pI_Cln_ in 1992 [116], ClC-3 in 1997 [117], and TMEM16F or anoctamin 6 (ANO6) in 2011 [118]. These candidates were, almost one after another, disproved by successive works performed in many laboratories (see [11] in detail), including our works [45, 62, 66, 70, 71, 82, 87, 119, 120]. Such a long time delay for molecular identification of VSOR/VRAC may be caused by the following four factors [31, 33]. First, ubiquitous expression of VSOR/VRAC activity in vertebrate cells precluded the expression cloning; second, all the available chemical inhibitors and activating ligands are not specific enough to perform the affinity purification; third, there exists no sequence homology among known chloride channel families; and fourth, the heterologous expression assay for a single candidate molecule must have been misleading, because the VSOR/VRAC channel turned out to be formed by multiple types, but not single type, of molecules, as described below.

### LRRC8 members as the core molecules

**Fig. 2:**
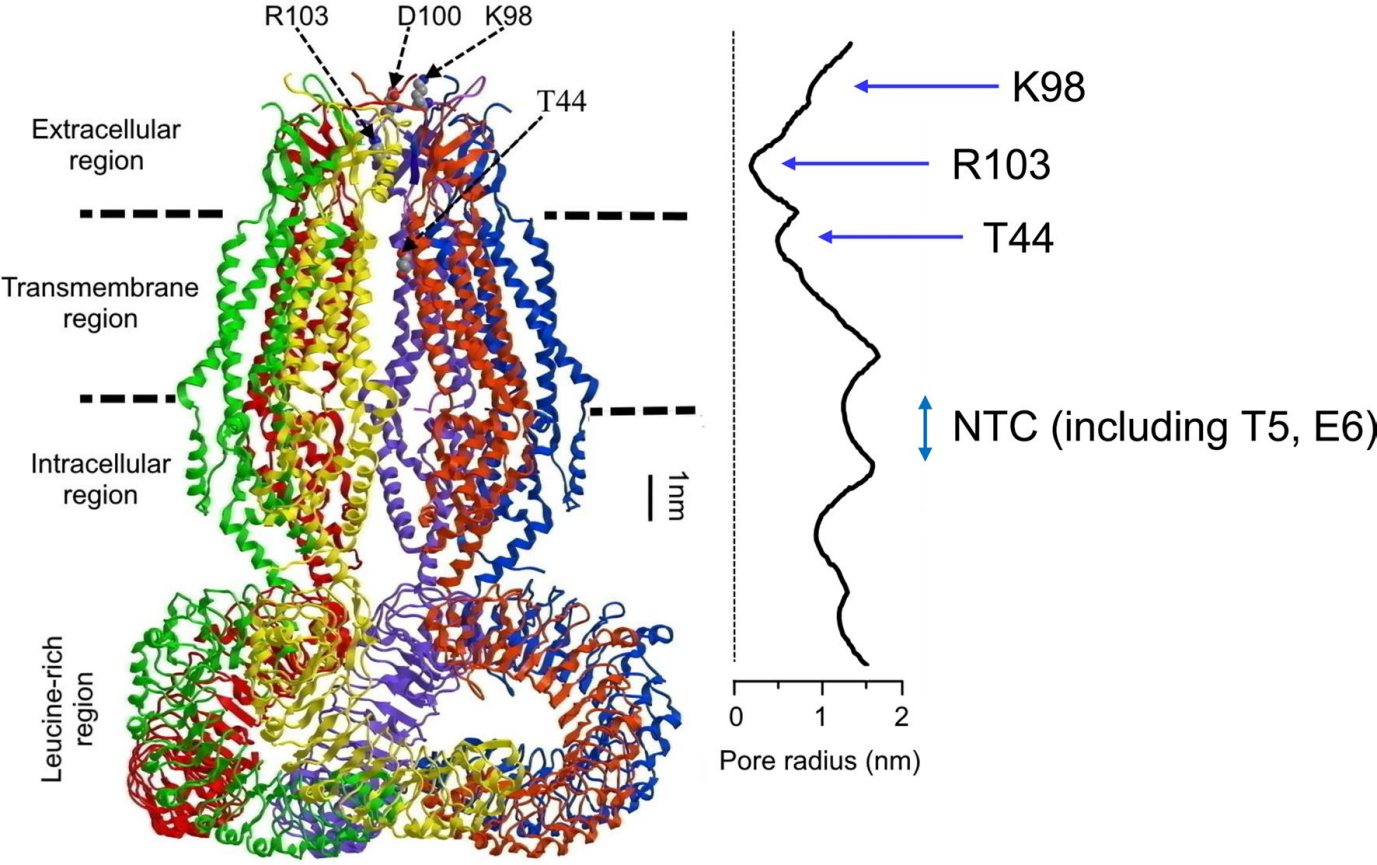
The side view of cryo-EM structure of the human LRRC8A homo-hexamer. The pore radius along the central axis is also depicted in scale. This figure is modified from Fig. 1 in [130] drawn according to [127]. NTC represents the *N*-terminal coil

Using an unbiased loss-of-function genome-wide RNAi screening method, LRRC8A was demonstrated to be a core molecule for VSOR/VRAC activity in human cells [8, 9] in 2014. Subsequently, a prerequisite role of LRRC8A in VSOR/VRAC activity was confirmed in mouse cells [40, 75, 121, 122], in rat cells [123, 124], in chicken cells [10], and in zebrafish cells [125]. Recent cryo-EM studies demonstrated that not only LRRC8A [126–130] but also another LRRC8 member, LRRC8D [131], form homo-hexameric structures consisting of the transmembrane pore domain, as depicted in Fig. 2 for LRRC8A. However, when overexpressed in cells deficient in all LRRC8 members, LRRC8A homomeric channels exhibited properties different from native VSOR/VRAC channels [132]; namely, insensitivity to osmotic cell swelling and only weak and voltage-dependent sensitivity to DCPIB, which is a known voltage-independent VSOR/VRAC channel blocker [100] and was shown to be most selective to other identified types of anion channel [34].

#### LRRC8A *plus* LRRC8C/D/E as the necessary components for VSOR/VRAC channels

The LRRC8 family is composed of five members of A to E with a molecular mass of around 95 kD consisting of around 800 amino acids. Since, collective gene disruption of LRRC8B, 8C, 8D, and 8E was found to abolish VSOR/VRAC activity [9, 133, 134], it is suggested that co-expression of LRRC8A with, at least, one of the other members is required for VSOR/VRAC activity. In fact, co-transfection of LRRC8A and LRRC8C, 8D, or 8E was found to rescue VSOR/VRAC activity in LRRC8^−/−^ HCT116 cells in which all five LRRC8 genes are disrupted [9]. Furthermore, co-immunoprecipitation studies provided evidence for the direct interaction between LRRC8A and LRRC8B/C/D/E in HEK293 cells [9] as well as between LRRC8D and LRRC8A/B/C in HEK293T cells, among LRRC8A, 8C, and 8D with each other in RAW264.7 macrophages, and among LRRC8A, 8B, and 8D with each other in KBM7 cells [135]. In addition, the trafficking of LRRC8B/C/D/E to the plasma membrane was detected when transfected with LRRC8A but not when transfected alone in HEK293 cells [9]. However, it appears that LRRC8B is not involved in the VSOR/VRAC formation in light of the following observations. First, when LRRC8B was co-transfected with LRRC8A, hypotonicity-induced VSOR/VRAC activity was never restored in LRRC8^−/−^ HCT116 cells [9]. Second, using LRRC8 members *C*-terminally tagged with fluorescent proteins, co-transfection of LRRC8A and LRRC8B failed to induce hypotonicity-induced VSOR/VRAC activity, whereas co-transfection of LRRC8A and LRRC8C/D/E produced the currents in enzymatically defolliculated *Xenopus* oocytes [136] in which endogenous VSOR/VRAC activity is lacking [137, 138]. Third, endogenous hypotonicity-induced VSOR/VRAC activity was abolished by triple knockdown of LRRC8C, 8D, and 8E in HeLa cells by Sato-Numata et al. [134] and by triple disruption of LRRC8C-8E genes in HEK293 cells by Lutter et al. [139] in 2017.

Most recent cryo-EM studies showed that LRRC8A and LRRC8C, in fact, assemble as a hetero-hexameric structure forming a pore domain [140, 141]. The number of hetero-hexameric complexes of LRRC8A and LRRC8C/D/E was estimated to be on the order of 10,000 by quantitative immunoblot studies [142], which is in good agreement with the functional VSOR/VRAC number estimated by the electrophysiological approach [49]. Mammalian cells may express different VSOR/VRAC channels simultaneously with different combinations of LRRC8A and LRRC8C/D/E. Previous co-immunoprecipitation studies suggested that the incorporation of only one LRRC8A subunit into a hetero-hexamer is sufficient for the formation of a functional VSOR/VRAC channel [142]. In contrast, most recent studies showed that VSOR/VRAC formed by LRRC8A *plus* LRRC8C exhibits 4:2 and 5:1 stoichiometry based on the data by mass spectrometry analysis [141] and those by single-particle cryo-EM analysis [140], respectively.

#### Questions about LRRC8A *plus* LRRC8C/D/E as the sufficient components for VSOR/VRAC channels

It is now obvious that LRRC8A and LRRC8C/D/E provide the necessary components for VSOR/VRAC channels. Then, it must be inquired whether LRRC8A and LRRC8C/D/E are sufficient components for the VSOR/VRAC entity in light of the following criteria for the molecular identification of the VSOR/VRAC channel [143]. Here, I update the criteria as follows: (*a*) The cells functionally exhibiting VSOR/VRAC activity should endogenously express the candidate mRNA(s) and protein(s); (*b*) the abolition of expression of the candidate protein(s) abolishes the endogenous VSOR/VRAC currents; (*c*) transfection with the gene(s) for the candidate protein(s) induces anionic currents with characteristics identical to those of phenotypic properties of VSOR/VRAC (Table 1: A, B); (*d*) the mutation(s) of the candidate gene(s) gives rise to significant changes in the biophysical pore properties of VSOR/VRAC (Table 1: B); and (*e*) reconstitution with the candidate protein(s) reproduces anionic currents with exhibiting the phenotypic properties of VSOR/VRAC (Table 1: A, B).

The fact that LRRC8 members are ubiquitously expressed in vertebrate cells [144] is in accord with Criterion-(*a*). In agreement with Criteria-(*b*), not only the single abolition of LRRC8A but also the quadruple abolition of LRRC8B/C/D/E were found to abolish the endogenous swelling-activated VSOR/VRAC currents [9, 132, 133]. Concerning Criterion-(*c*), combined overexpression of LRRC8A and LRRC8C/D/E was shown to induce swelling-induced outwardly rectifying anion currents in LRRC8^−/−^ HCT116 cells [9] and defolliculated *Xenopus* oocytes [136]. However, it has not been examined as yet whether these anion currents exhibit the full set of phenotypic properties of VSOR/VRAC (Table 1), especially including non-hydrolytic dependence on cytosolic ATP, sensitivity to cytosolic free Mg^2+^, and low-field Eisenman type-I anion selectivity. Regarding Criterion-(*d*), a number of point mutation studies have been performed, as given below, on the whole-cell current level, but not on the single-channel level as yet. Outward rectification of whole-cell VSOR/VRAC currents was found to become stronger when the T5R mutants of LRRC8A and LRRC8C were expressed in LRRC8^−/−^ HCT116 cells [145], suggesting that the short hydrophilic *N*-terminal coil (NTC) region (Fig. 2) is important for the outward-rectifying properties of VSOR/VRAC. The time course of inactivation kinetics of whole-cell currents observed at large positive potentials was found to be affected by charge-modifying mutation of K98 and D100 of LRRC8A (Fig. 2) as well as the corresponding residues (K91 and N93) of LRRC8E in the *C*-terminal part of the first extracellular loop (EL1), when these mutants of LRRC8A and LRRC8E were both expressed in LRRC8^−/−^ HCT116 cells [145], as follows. The inactivation kinetics becomes much faster by combinations of charge-reversing K98E-LRRC8A and K91E-LRRC8E mutations as well as by those of D100R- or D100K-LRRC8A and N93R- or N93K-LRRC8E mutations. In contrast, the kinetics becomes slower by a combination of charge-neutralizing K98A- or K98N-LRRC8A and K91A- or K91N-LRRC8E mutations as well as by a combination of charge-preserving D100E-LRRC8A and charge-conferring N93E-LRRC8E mutations. In addition, the voltage dependence of the inactivation kinetics was found to be observed at much lesser positive potentials by a combination of charge-neutralizing E6C-LRRC8A and -LRRC8E mutations in the NTC domain. Since the positively charged R103 residue in the extracellular subdomain of LRRC8A forms the narrowest constriction (Fig. 2), the mutation of this residue may be predicted to affect the pore properties of VSOR/VRAC. In fact, the introduction of a charge-neutralizing R103F mutation into LRRC8A was found to abolish the open-channel blocking action of extracellular ATP, when co-expressed with wild-type LRRC8C [128]. Also, the anion selectivity of LRRC8A + LRRC8C channels was shown to be reduced by this mutant of LRRC8A [128]. The introduction of another charge-neutralizing R103A mutation into LRRC8A rendered the LRRC8A + LRRC8C channel slightly permeable to Na^+^ by changing the P_Na_/P_Cl_ value from 0.02 to 0.28 [126]. In contrast, the positive charge-conferring L105R mutation of the corresponding residue of LRRC8C failed to change the anion selectivity of LRRC8A + LRRC8C channel [126]. Another positively charged K98 residue, existing near R103, in the EL1 domain of LRRC8A (see Fig. 2) appears to be also involved in the anion selectivity filter because the introduction of double charge-reversing K98E and K91E mutations to LRRC8A and LRRC8E, respectively, decreased the P_I_/P_Cl_ value from 1.25 to 1.12 [146]. In addition, it is likely that T5 and E6 in the NTC domain (see Fig. 2) are involved in the anion selectivity because the channels formed by T5R-LRRC8A together with E6C-LRRC8C and by E6C-LRRC8A together with E6C-LRRC8C or E6C-LRRC8E exhibited increased the P_I_/P_Cl_ value from 1.39 to 1.83 [128] and from 1.29 to 2.29 [145], respectively. However, these charge-modified point mutants unfortunately exhibited the same sequence of P_I_ > P_Cl_ selectivity though in varying degrees. To assert that the above-studied residues form the anion selectivity filter, much stronger changes in the selectivity sequence, such as reversal of the P_I_: P_Cl_ sequence, that is, transition of types of Eisenman’s halide anion selectivity sequence from type I to types III – VII (Table 2) or just transition of types of the halide anion selectivity sequence from type I to type II (Table 2), are to be observed by the mutational studies by comparing permeabilities to I^−^ and Cl^−^ to Br^−^ at least. However, it must be pointed out that such mutational studies must be performed not only on the whole-cell current level but also on the single-channel level to see the effect on the intermediate outwardly rectifying unitary conductance, in the future. Taken together, these mutational studies indicate that LRRC8A *plus* LRRC8C/D/E may form the pore of VSOR/VRAC, although the crucial evidence is still missing. Moreover, no mutational studies have been performed as yet for intracellular ATP dependence and intracellular Mg^2+^ sensitivity.

#### Requirements of some unidentified additional components for VSOR/VRAC channels

To obtain more direct evidence for the involvement of LRRC8 members, reconstitution studies were recently conducted [127, 129, 133]. The anion channels reconstituted with LRRC8A alone were found to exhibit completely different properties from those of native VSOR/VRAC channels. LRRC8A channels reconstituted in liposomes exhibited homo-hexameric structure and are activated only by reduction of ionic strength in a manner totally independent of ATP [127, 129] and insensitive to Mg^2+^ [129]. In addition, the unitary conductance of LRRC8A channels looks smaller and more sharply outwardly rectifying than that of native VSOR/VRAC channels; that is, around 50 pS at + 100 mV in 500 mM Cl^−^ conditions [127] and 24 pS at + 100 mV and 5.4 pS at − 100 mV in 70 mM Cl^−^ conditions [129]. When LRRC8A and LRRC8C, 8D, or 8E were reconstituted to form a hetero-hexameric channel in droplet lipid bilayers, a strong hypotonic stimulation (around 47 ~ 77% osmolarity) was found to steadily activate anionic single-channel events, even in the absence of ATP, exhibiting intermediate, but relatively small, unitary conductance of around 8 pS and 50–70 pS in the presence of 70 mM and 500 mM KCl, respectively [133]. However, no single-channel-level studies on the hetero-hexameric channels reconstituted with LRRC8A and LRRC8C/D/E have so far been conducted concerning outward rectification on the single-channel level as well as anion selectivity sequence, Mg^2+^ sensitivity, and voltage-dependent blocking by ATP on the whole-cell level. Moreover, the unitary events were steadily observed at + 100 mV without exhibiting inactivation kinetics and could be activated preferably by reduction of ionic strength in the absence of ATP in both *cis* and *trans* solutions [133]. Above all, the channels were never activated by swelling (droplet inflation) per se [133], in contrast to native VSOR/VRAC channels [46–48]. Thus, LRRC8A *plus* LRRC8C/D/E form anionic channels in the cell-free reconstituted system in a manner completely different from native VSOR/VRAC channels observed in the cell system even though it appears that they participate in the pore formation for the molecular entity of VSOR/VRAC.

### Recently proposed molecules required for VSOR/VRAC channel activities

Collectively, it must be concluded that the recent mutational and reconstitution studies with LRRC8 members, unfortunately, failed to alter and reproduce, respectively, all the full set of phenotypical properties of native VSOR/VRAC channels. Also, we here need to remind the following important facts. First, overexpression of LRRC8A alone suppressed endogenous VSOR/VRAC activity [8, 9]. Second, overexpression of LRRC8A *plus* LRRC8C failed to increase VSOR/VRAC currents by adding new currents to the endogenous currents [9]. Third, cisplatin-resistant KCP-4 cells deficient in endogenous VSOR/VRAC activity exhibit similar protein expression levels of LRRC8 members to those in the parental KB cells as well as in other three (HEK293T, HeLa, and Intestine 407) human epithelial cell lines rich in VSOR/VRAC activity [11, 75]. Fourth, overexpression of LRRC8A *plus* LRRC8D or LRRC8E in VSOR/VRAC-deficient KCP-4 cells failed to restore VSOR/VRAC activity up to that observed in its parental KB cells [75]. Thus, it is evident that we still miss some as-yet-unidentified additional essential component(s) other than LRRC8 members for VSOR/VRAC activity exhibiting a full set of phenotypical properties (Table 1).

#### TTYH hypothesis

Following the recent LRRC8 upsurge, two groups [147, 148] proposed TTYH members, the genes of which are homologs of the *Drosophila melanogaster tweety*, as the pore-forming proteins of VSOR/VRAC in 2019. TTYH1 was initially described as a large-conductance Maxi-Cl anion channel activated by hypotonic stimulation [149, 150] but later this hypothesis was disproven [10, 63]. Han et al. [147] originally reported that TTYH members serve as the core components of VSOR/VRAC channel based on the following observations. First, shRNA-mediated knockdown of LRRC8A and/or LRRC8C failed to abolish I_Cl,swell_ in primary mouse astrocytes equilibrated with intracellular (pipette) and extracellular Tris-Cl-rich solutions, although the same knockdown procedure abolished VSOR/VRAC currents in HEK293 cells equilibrated with NaCl-rich solutions. Second, shRNA-mediated triple knockdown of TTYH1, 2, and 3 mostly abolished I_Cl,swell_ in mouse astrocytes in primary culture and those in hippocampal slices under Tris-Cl-rich conditions. Third, overexpression of TTYH1, 2, and 3 together with water channel AQP4 enhanced I_Cl,swell_ in HEK293 cells under Tris-Cl-rich conditions and rescued the currents in HEK293 and CHO-K1 cells both in which LRRC8A expression was knocked down by treatment with shRNA-LRRC8A. Furthermore, they concluded that TTYH1/2/3 forms the pore of VSOR/VRAC channels because overexpression of the charge-neutralized R165A-TTYH1 and R164A-TTYH2 mutants suppressed I_Cl,swell_ in HEK293 and CHO-K1 cells, respectively, under Tris-Cl-rich conditions, and, in addition, because extracellular application of MTSES did not affect I_Cl,swell_ recorded in HEK293 cells transfected with WT-TTYH1 *plus* AQP4 but became suppressive against the currents recorded in those transfected with R165C-TTYH1 *plus* AQP4 under Tris-Cl-rich conditions. About 1 month later, Bae et al. [148] also deduced that TTYH1 and TTYH2 confer VSOR/VRAC in a manner independent of LRRC8A in gastric cancer cells under the intracellular and extracellular Tris-Cl-rich conditions based on the following observations. First, I_Cl,swell_ was not affected by shRNA-mediated knockdown of LRRC8A but, in contrast, was largely abolished by double knockout of TTYH1 and TTYH2 in gastric cancer SNU-601 cells. Second, expression of TTYH1/2, but not LRRC8A, mRNA is lacking in cisplatin-resistant SNU-601-derived R10 cells in which VSOR/VRAC activity is deficient, but I_Cl,swell_ and TTYH1/2 mRNA expression were both restored after treatment with a histone deacetylase inhibitor TSA. Third, the knockdown of TTYH1 alone and that of TTYH2 alone inhibited I_Cl,swell_ in HepG2 cells which endogenously express TTYH1 but not TTYH2, and in LoVo cells which endogenously express TTYH2 but not TTYH1, respectively. However, it must be noted that a small but significant level of I_Cl,swell_ was still observed even after triple knockdown of TTYH1, 2, and 3 in astrocytes [147] and double knockout of TTYH1 and TTYH2 in SNU-601 cells [148]. Moreover, I_Cl,swell_ was actually suppressed by knockdown of LRRC8A and/or LRRC8C in HEK293 cells under NaCl-rich conditions [147]. Thus, it is possible that a membrane-impermeable cation, Tris^+^, somehow impairs the knockdown of LRRC8 expression, and then TTYH blocks this Tris^+^ effect.

Recent cryo-EM studies provided firm evidence against “TTYH = VSOR/VRAC” hypothesis. First, no pore-like structure was observed in the three-dimensional structure of TTYHs [151, 152], which were shown to be expressed in the plasma membrane [153]. The positively charged residues, such as R165 of TTYH1, R164 of TTYH2 as well as R367 and H370 of TTYH3, that were previously proposed to line a pore [147, 149], exist remote from the membrane [152]. Also, the electrostatic environment at the extracellular side within the transmembrane domain is formed by strongly acidic (negatively charged) residues [151, 152] that should repel permeating anions. Second, hypotonicity-induced VSOR/VRAC-like currents were never observed even under Tris-Cl-rich conditions in HEK293 cells in which TTYH2 alone or together with AQP4 was overexpressed [151], and also never observed under NMDG-Cl-rich conditions in LRRC8^−/−^ HEK293 cells, in which TTYH1, 2, or 3 together with AQP4 were overexpressed [152]. Above all, although expression of TTYH proteins was found to be exclusively in astrocytes in the mouse hippocampus and cortex [147], vigorous VSOR/VRAC activity was actually observed in neurons in the mouse [154] and rat [106, 155] hippocampus as well as in neurons in the mouse cortex [59, 76]. Taken together, it is conceivable that TTYHs represent neither VSOR/VRAC molecules nor its essential core components.　However, there remains a possibility that TTYHs play augmenting roles in VSOR/VRAC channel activity only under special (such as Tris-Cl-rich) conditions in some (but not all) cell types including astrocytes and gastric cancer cells.

#### TRPM7 as the swelling-sensing subcomponent

The physiologically most important phenotype of the VSOR/VRAC is volume expansion sensitivity. During the RVD process, this anion channel participates in the volume-regulatory Cl^−^ efflux [3, 4, 14, 29]. The channels reconstituted with LRRC8A *plus* LRRC8C/D/E were, however, not activated by cell swelling, though activated by a reduction in the ionic strength [133]. Thus, the volume sensitivity of VSOR/VRAC must be granted by some essential subcomponent other than LRRC8 members. The channel-mediated volume-regulatory KCl efflux was shown to be preceded by the activation of a Ca^2+^-permeable mechano-sensitive cation channel [156–158], which was recently identified as TRPM7 in human epithelial cells [159]. Membrane stretch-induced activation of TRPM7 was demonstrated to cause Ca^2+^ influx, thereby stimulating Ca^2+^-activated K^+^ channels (K_Ca_) [159], that are responsible for volume-regulatory K^+^ efflux [1, 160]. In light of such a close coupling of VSOR/VRAC, K_Ca_, and TRPM7 functions in the RVD process, there arises a possibility that TRPM7 expressed ubiquitously is involved in the activation not only of K_Ca_ but also of VSOR/VRAC. Recently, Numata et al. [10], in fact, provided firm evidence that TRPM7 functionally regulates VSOR/VRAC activity by molecularly interacting with LRRC8A and by sensing volume expansion, as follows. First, shRNA-mediated knockdown of TRPM7 prominently reduced both swelling-activated VSOR/VRAC currents and human LRRC8A mRNA expression in HeLa cells. In addition, the knockout of gallus TRPM7 (gTRPM7) abolished both VSOR/VRAC currents and gLRRC8A mRNA expression in chicken DT40 cells. Since 2 day treatment with a TRPM7 blocker NS8593 or a Ca^2+^ chelator EGTA was found to suppress not only TRPM7 currents and the cytosolic Ca^2+^ level but also VSOR/VRAC currents, it appears that the regulatory effect of TRPM7 on VSOR/VRAC activity is mediated by steady-state Ca^2+^ influx through TRPM7 channels. Second, plasmalemmal colocalization and physical interaction between LRRC8A and TRPM7 were observed by immunostaining and coimmunoprecipitation studies, respectively, in osmotically swollen HeLa cells. Such colocalization and physical interaction were also observed between LRRC8A and the K1648R construct of TRPM7, in which enzyme activity of the *C*-terminal α-kinase domain was inactivated by the point mutation of the ATP-binding site, but were never observed between LRRC8A and the Δ-kinase construct of TRPM7, in which the entire α-kinase domain was deleted. Third, it appears that VSOR/VRAC activity is exhibiting a functional coupling, in real-time, with TRPM7 activity, because linear relationships were observed between TRPM7-mediated cationic current densities and VSOR/VRAC-mediated anionic current densities simultaneously measured after hypotonic stimulation in the same DT40 cells as well as in the same gTRPM7-deficient DT40 cells complemented with wild-type hTRPM7. Taken together, it is concluded that TRPM7 serves as an essential volume-sensitive subcomponent of VSOR/VRAC first by enhancing LRRC8A mRNA expression via steady-state Ca^2+^ influx, second by stabilizing the plasmalemmal expression of LRRC8A protein via physical interaction, and third by exhibiting a real-time functional coupling with VSOR/VRAC activity.

## Conclusions and perspectives

The volume-sensitive/regulatory outwardly rectifying anion channel, VSOR/VRAC, functionally discovered in 1988 exhibits phenotypical properties such as volume expansion sensitivity, cytosolic ATP dependence, intracellular Mg^2+^ sensitivity, and low-field anion selectivity, those of which are distinct from other anion channels. Unfortunately, specific VSOR/VRAC blockers are not available as yet and are to be surveyed or developed by taking two basic structures of known effective blockers into consideration. Therefore, one could not perform a search for the ubiquitous VSOR/VRAC molecule by affinity purification using any specific inhibitors. By an unbiased genome-wide siRNA screening approach, then, the hetero-multimeric complex of LRRC8A and LRRC8C/D/E was identified as the core, highly possibly pore-forming, component of VSOR/VRAC in 2014. However, the LRRC8 channel per se was shown to be insensitive to osmotic swelling but activated by the reduction of ionic strength even in the absence of cytosolic ATP. Recently, a Ca^2+^-permeable mechano-sensitive non-selective cation channel TRPM7 was identified as the essential subcomponent that endows the anionic VSOR/VRAC channel with volume expansion sensitivity. It must be emphasized that some other subcomponent(s) giving cytosolic ATP dependence and Mg^2+^ sensitivity are still missing. Thus, studies still need to keep in progress to identify the molecular entity of VSOR/VRAC channels exhibiting the full set of phenotypical properties.

From not only physiological but also pathological and clinical perspectives, elucidation of the activation mechanisms and the roles in release of organic signals and in cell death induction of VSOR/VRAC is very important. In this regard, the molecular entity of VSOR/VRAC is to be reassessed. These topics will be described in the subsequent Part 2 article.

## Data Availability

The data underlying this article will be obtained via PubMed and Google Scholar or available from the author upon reasonable request.

## Abbreviations

RVD: Regulatory volume decrease
CaCC: Ca^2+^-activated Cl^−^ channel
VSOR: Volume-sensitive outwardly rectifying anion channel
VRAC: Volume-regulated anion channel
OVD: Osmotic volume decrease
OVI: Osmotic volume increase
HVD: Hormonal volume decrease
HVI: Hormonal volume increase
SVD: Secretory volume decrease
CVD: Cooling volume decrease
FVD: Freezing volume decrease
RVI: Regulatory volume increase
NKCC: Na^+^-K^+^-Cl^−^ symporter or cotransporter
NCC: Na^+^-Cl^+^ symporter
NHE: Na^+^/H^+^ antiporter or exchanger
AE: Cl^−^/HCO_3_^–^ antiporter (anion exchanger)
KCC: K^+^-Cl^−^ symporter
KHE: K^+^/H^+^ antiporter
HICC: Hypertonicity-induced cation channel
EC_50_: Half-maximal effective concentration
IC_50_: Half-maximal inhibitory concentration
ASOR: Acid-sensitive outwardly rectifying anion channel
PAC: Proton-activated anion channel
I_Cl,swell_: Swelling-activated Cl^−^ currents
CysLT1: Cysteinyl leukotriene 1
PGP: P-glycoprotein
ANO6: Anoctamin 6
EL1: First extracellular loop
NTC: *N*-Terminal coil
K_Ca_: Ca^2+^-activated K^+^ channel
